# Three epitope-distinct human antibodies from RenMab mice neutralize SARS-CoV-2 and cooperatively minimize the escape of mutants

**DOI:** 10.1038/s41421-021-00292-z

**Published:** 2021-07-20

**Authors:** Jianhui Nie, Jingshu Xie, Shuo Liu, Jiajing Wu, Chuan Liu, Jianhui Li, Yacui Liu, Meiyu Wang, Huizhen Zhao, Yabo Zhang, Jiawei Yao, Lei Chen, Yuelei Shen, Yi Yang, Hong-Wei Wang, Youchun Wang, Weijin Huang

**Affiliations:** 1 Division of HIV/AIDS and Sex-transmitted Virus Vaccines, Institute for Biological Product Control, National Institutes for Food and Drug Control (NIFDC) and WHO Collaborating Center for Standardization and Evaluation of Biologicals, Beijing, China; 2 Beijing Biocytogen Co., Ltd, Beijing, China; 3 Shuimu BioSciences Co., Ltd, Beijing, China; 4 Ministry of Education Key Laboratory of Protein Sciences, Tsinghua-Peking Joint Center for Life Sciences, Beijing Advanced Innovation Center for Structural Biology, School of Life Sciences, Tsinghua University, Beijing, China

**Keywords:** Cryoelectron microscopy, Pattern recognition receptors

## Abstract

Coronavirus disease 2019 (COVID-19), a pandemic disease caused by the newly emerging severe acute respiratory syndrome coronavirus 2 (SARS-CoV-2), has caused more than 3.8 million deaths to date. Neutralizing antibodies are effective therapeutic measures. However, many naturally occurring mutations at the receptor-binding domain (RBD) have emerged, and some of them can evade existing neutralizing antibodies. Here, we utilized RenMab, a novel mouse carrying the entire human antibody variable region, for neutralizing antibody discovery. We obtained several potent RBD-blocking antibodies and categorized them into four distinct groups by epitope mapping. We determined the involved residues of the epitope of three representative antibodies by cryo-electron microscopy (Cryo-EM) studies. Moreover, we performed neutralizing experiments with 50 variant strains with single or combined mutations and found that the mixing of three epitope-distinct antibodies almost eliminated the mutant escape. Our study provides a sound basis for the rational design of fully human antibody cocktails against SARS-CoV-2 and pre-emergent coronaviral threats.

## Introduction

The wild spread of severe acute respiratory syndrome coronavirus 2 (SARS-CoV-2) has caused more than 100 million infections, resulting in 3.8 million reported deaths until June 13, 2021. SARS-CoV-2 is a highly contagious enveloped positive-strand RNA virus of the betacoronavirus genus^1^. The viral infection is established by hijacking the cell surface receptor for entry. In the case of SARS-CoV-2, the spike (S) protein forming a large homotrimeric complex at the viral surface is required for this role. The structure of the S protein trimer has been well characterized^2^. Similar to that of severe acute respiratory syndrome coronavirus (SARS-CoV), it is heavily glycosylated and each protomer consists of an N-terminal domain (NTD), a receptor-binding domain (RBD), and an S2 domain. Each RBD at the top of the complex can adopt either open (up) or closed (down) conformation. An open RBD is critical for hooking up potential receptors, membrane fusion, and subsequent viral entry. Although several plasma membrane proteins have been shown to interact with S protein^3^, human angiotensin-converting enzyme 2 (hACE2) is believed to be the major human receptor hijacked by the virus^4^. So far, blocking RBD–hACE2 engagement is the primary target of most preventative and therapeutic measures^5^.

Most groups have taken advantage of convalescent coronavirus disease 2019 (COVID-19) patients for fully human antibody discovery. The peripheral blood mononuclear cells (PBMCs) of these people are amenable for collection due to a large number of clinical cases. RBD-specific B cells can be enriched and subjected to single-cell analysis to gain paired antibody chain sequences. On the other hand, high-affinity fully human antibodies can be obtained from transgenic human antibody mice, which represent a valuable alternative. For example, Regeneron’s COVID-19 antibody cocktail (REGN-COV2) contains two fully human immunoglobulin G1 (IgG1) antibodies, one selected from the Velocimmune mouse platform and the other isolated from human subjects^6^. Similarly, some cross-neutralizing antibodies for SARS-CoV-2 and the related SARS-CoV were identified from human PBMCs (S309)^7^, normal mouse (H014)^8^, and transgenic H2L2 mouse (47D11)^9^.

A novel fully human antibody mouse named RenMab was recently developed by Biocytogen (www.renmab.com). In this novel mouse model, the 2.6 Mb (megabase) heavy chain and 3.2 Mb kappa chain sequence of mouse antibody variable regions (from the first 5' V gene to the last 3' J gene) was replaced by the entire human variable region segments, while the mouse constant regions were untouched. This mouse mounted robust antibody responses upon immunization of various foreign antigens. In this study, we screened SARS-CoV-2 RBD-specific antibodies from RBD-immunized RenMab mice and found that ten antibodies efficiently blocked hACE2 binding. These antibodies bound RBD with affinity in the nanomolar (nM) range and exhibited good neutralizing activity against pseudotyped viruses. Recently, many spontaneous mutations have been identified to accumulate in clinical samples. These mutations, e.g., D614G, showing increased infectivity, pose a significant concern to the field^10^. Mutations that occurred in the RBD region have more chances to affect hACE2 binding and evade existing antiviral therapies^10^. For example, a single amino-acid mutation has been found to fully escape both antibodies in the REGN-COV2 cocktail^11^. Using a competitive binding test, we found that RBD-blocking antibodies can be separated into multiple bins. Further, we performed cryo-electron microscopy (Cryo-EM) structure studies of the spike–Fab complex of three representative antibodies. Structure–function correlation was established by screening individual antibodies or in combination against a pseudotyped virus library containing 35 single mutations and 15 combined mutations in SARS-CoV-2 S protein. The three selected antibodies were found to have significant reductions in neutralization to 20, 5, and 10 variants, respectively. However, the escape can be reduced by applying two or three epitope-distinct antibodies. Our study supports the notion that SARS-CoV-2 mutant variants’ escape from a single neutralizing antibody is common and inevitable^12^. Given the nature of infectious agents, the rationale combination of multiple antibodies with distinct structural epitopes is considered to be a reasonable solution for COVID-19 therapy.

## Results

### Generation and screening of SARS-CoV-2 RBD-specific antibodies from RenMab mice

We used recombinant SARS-CoV-2 RBD as an immunogen and hACE2 blocking activity for initial screening. A cohort of RenMab mice were immunized subcutaneously, and the antibody titer was quantified by staining with CHO cells ectopically expressing S protein. Most mice exhibited strong serum reactivity at a titer over 1:25,600 dilution, and we selected five mice to perform hybridoma fusion. From about 2000 wells, we found that more than 500 wells showed variable degrees of blocking activity in a cell-based assay. Subcloning was carried out for 209 wells, and we generated ten stable hybridoma clones. We confirmed that these clones bound to S protein and competed with soluble hACE2 (Supplementary Fig. S1). Paired heavy- and light-chain DNA sequences of these ten clones were successfully retrieved by 5' rapid amplification of cDNA ends (RACE)-based PCR, sequenced, and cloned into a human IgG1-expressing vector. Recombinant antibodies were made in ExpiCHO-S cells. All recombinant antibodies bound to RBD with affinity at nanomolar or lower range (Supplementary Table S1 and Fig. S2). We loaded RBD on a chip, and the subsequent flow in hACE2 over the chip caused a significant signal increase, indicating the hACE2–RBD interaction. Preincubating with antibodies strongly reduced the signal of hACE2 binding (Supplementary Fig. S3). Recent studies suggested that certain immunoglobulin-heavy variables (IGHVs), especially those in the IGHV3 family, are preferentially enriched in RBD-specific antibodies recovered in humans^6,10,13^. Interestingly, we found that six of ten clones used IGHV3 genes, while the other four clones used IGHV4 (Supplementary Table S1), which is consistent with recent studies that a strong IGHV3 usage bias was found in the anti-SARS-CoV-2 antibody sequence repertoire in humans. Taken together, diverse fully human antibodies with a high affinity toward the RBD domain on viral S protein were discovered and they exhibited potent blocking activities in both cell-based and cell-free assays.

### Binding characteristics of four types of RBD-blocking antibodies

**Fig. 1:**
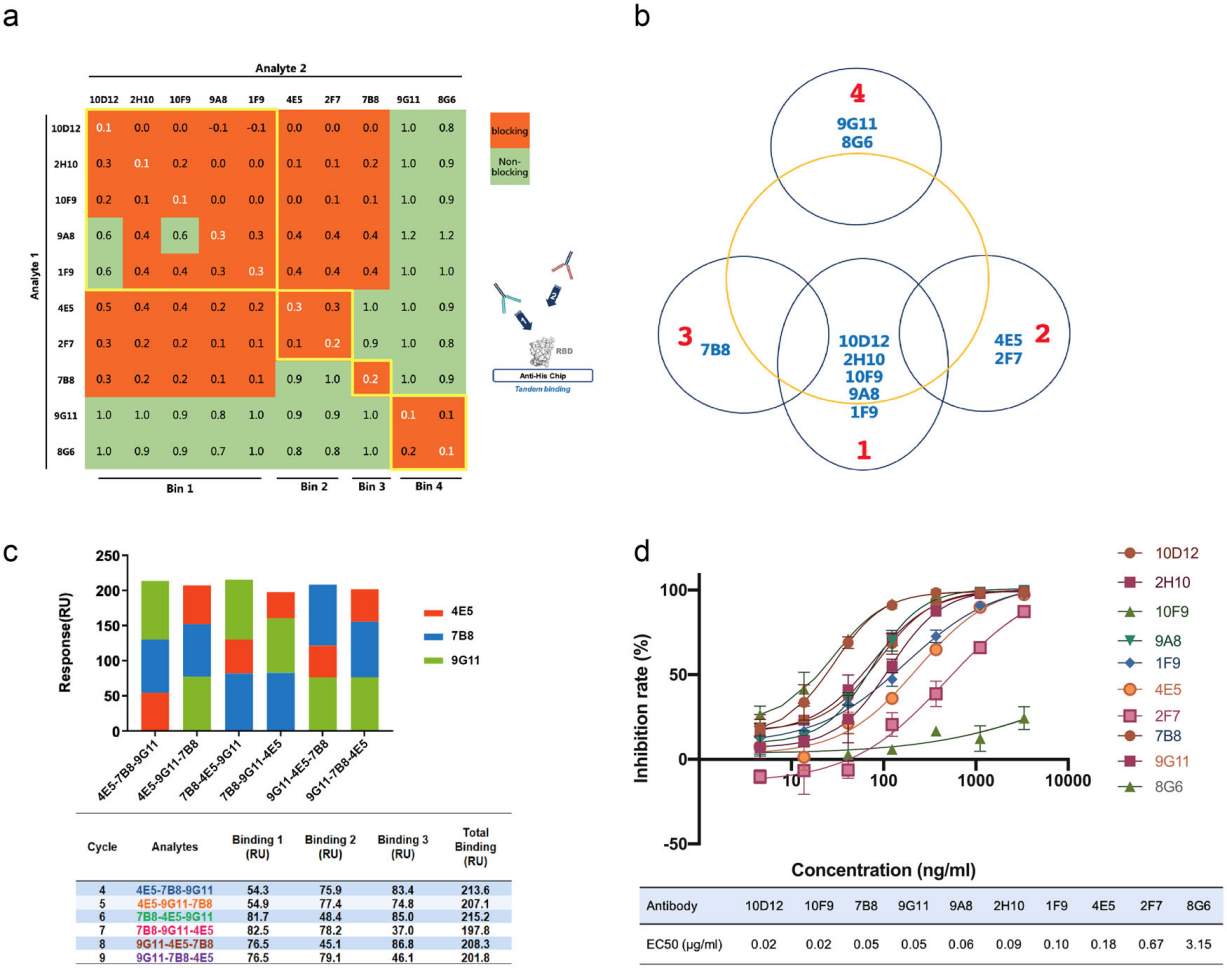
Separable binding interfaces of a group of hACE2-blocking antibodies derived from RenMab mice. **a** The relative positions of epitopes on the SARS-CoV-2 RBD domain where ten blocking antibodies could bind were examined by a sequential flow of any two antibodies (in all combinations) over the biosensor surface-immobilized with SARS-CoV2 RBD and evaluated by an index, which was calculated as the ratio of RU (analyte 2 in the absence of analyte 1) to RU (analyte 2 in the presence of the indicated analyte 1). Boxes with a score of 0.6 or greater were colored green, and those with a score smaller than 0.6 were colored orange. The clones that belong to the same bin were indicated by the area enclosed by yellow lines. **b** Schematic diagram of the relative positions of four distinct bins of antibodies. The yellow circle represents the hACE2-binding interface on SARS-CoV-2 RBD. **c** Simultaneous binding by three nonoverlapping antibodies. SARS-CoV2-RBD was immobilized on a biosensor as bait. 4E5, 7B8, and 9G11 flowed into the system sequentially in the indicated order. **d** hACE2-blocking antibodies efficiently neutralize the pseudotyped virus in cell culture. Five representative antibodies derived from RenMab mice were first incubated with SARS-CoV-2 (WH01) pseudotyped virus at the titrated concentration, then the mixtures were applied to Huh-7 cells. The neutralizing activity was visualized by plotting the luciferase signal after 24 h against the antibody concentration.

Given the sequence diversity of antibodies, we sought to determine their relative binding positions on RBD. Epitope binning was carried out to study the extent of competition between different clones by sequential flow in a pair of antibodies over an RBD-loaded chip. Interestingly, we detected at least four epitope-distinct clusters (Fig. 1a). Bin 1 is the largest cluster including five clones (10D12, 2H10, 10F9, 9A8, and 1F9), whose heavy chains belong to the IGHV3 family. Importantly, four of them contained the NY motif in the complementarity-determining region (CDR) H1 and SGGS motif in the CDR H2, which are the key structural determinants for a group of SARS-CoV-2-neutralizing antibodies^14^. The remaining clones can be sorted into three nonoverlapping bins. Bin 2 has two clones (4E5 and 2F7), Bin 3 has one clone (7B8), and Bin 4 has two clones (9G11 and 8G6). Importantly, the data suggest that the neutralizing antibodies targeting the interface have several interesting patterns. First, the binding of Bin 4 antibodies did not affect that of any other Bin, and vice versa, indicating that the epitopes of 9G11 and 8G6 have no overlapping with other Bins. Second, Bin 2 and Bin 3 epitopes are physically separated, while they both showed interference with Bin 1. On the basis of this information, a binding pattern showing the epitopes of ten neutralizing antibodies was depicted (Fig. 1b). Bins 2, 3, and 4 were put at the three vertexes of a triangle covering the hACE2-binding interface on RBD. Bin 1 epitope stood in between Bin 2 and 3. Though all four bins are predicted to invade the hACE2 interface, Bin 4 alone is on one of two sides. To show whether this triangle topology can be supported by experimental validation, we selected 4E5 (Bin 2), 7B8 (Bin 3), and 9G11 (Bin 4) to represent the three nonoverlapping Bins. We performed triple competition assays on the RBD-immobilized chip by flowing three antibodies in six combined orders. Consistent with our model, these antibodies yielded a comparable signal when they flew in queues or flow alone, suggesting that a single RBD protein can be simultaneously bound by the three antibodies with almost no steric hindrance (Fig. 1c). Our data, along with others, suggested that RBD itself contains several physically separable neutralizing epitopes, supporting the notion of a rational combination of different clones to achieve better blocking. To determine whether these antibodies were capable of blocking virus entry, their neutralizing ability was tested with wild-type (WT) S protein (Wuhan strain WH01) pseudotyped VSV with a luciferase reporter. Preincubation of the virus with these antibodies led to significant reductions of luciferase reporter gene expression in cell culture in a concentration-dependent manner (Fig. 1d). Interestingly, the neutralizing potency comparison showed 10D12 = 10F9 > 7B8 = 9G11 > 9A8 > 2H10 > 1F9 > 4E5 > 2F7 > 8G6. The half-maximal effective concentration (EC_50_) values for 10D12, 7B8, and 9G11 were 0.02, 0.05, and 0.05 μg/mL, respectively. Although Bin 2 antibodies (4E5 and 2F7) are located in a relatively separated epitope, their neutralizing potencies were much lower, for which the EC_50_ values were 0.18 and 0.67 μg/mL, respectively. Therefore, we chose the antibodies 10D12, 7B8, and 9G11 for the following investigation.

### Structure analysis of three representative neutralizing antibodies

**Fig. 2:**
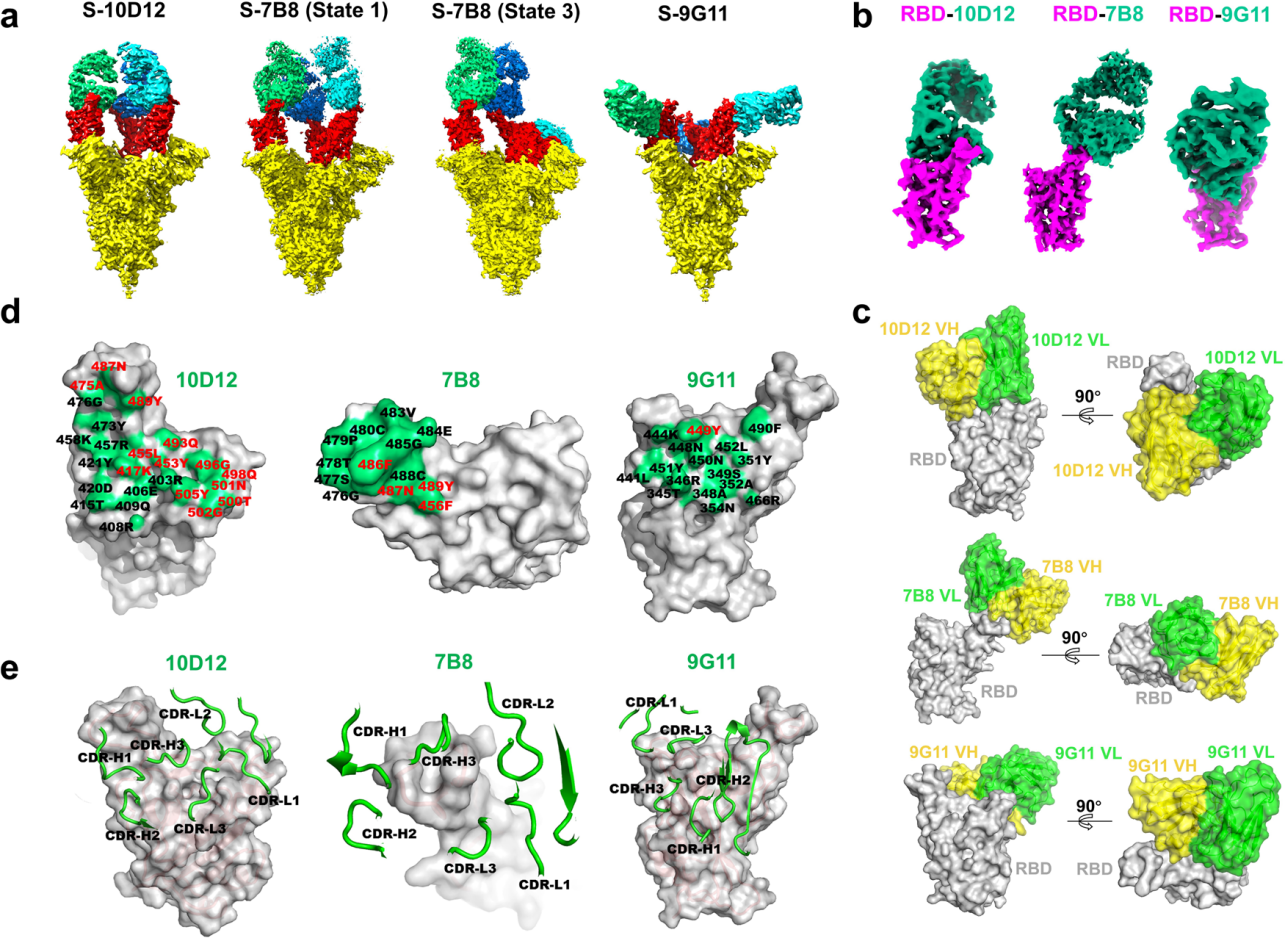
Cryo-EM structures reveal the binding interface between the three neutralizing antibodies and the SARS-CoV-2 S trimer. **a** Overall structures of trimeric SARS-CoV-2 S protein in complex with three antibodies. The RBD domain is shown as red, and Fab is shown as cyan/blue/green. **b** The cryo-EM map of a single Fab fragment bound to RBD after local refinement. The RBD domain is shown as magenta, and Fab is shown as green. **c** Side (left) and top (right) views of the cartoon representation of antibody VH (yellow surface) and VL (green surface) bound to RBD (gray surface). **d** The footprint of Fab on SARS-CoV-2 RBD is colored as a green surface and the corresponding epitopes on RBD are labeled as red (overlapped with hACE2) or black (not overlapped with hACE2). **e** CDR loops of Fabs overlaid on a surface representation of the RBD (gray surface) show the interaction between Fab and RBD.

Each RBD in the trimeric S complex can adopt either open or closed conformation, which regulates the accessibility by hACE2, as well as neutralizing antibodies. The combined data of epitope binning and virus-neutralizing ranking promoted us to select three antibodies, namely 10D12, 7B8, and 9G11, for structure elucidation. Intact monoclonal antibody (mAb) (for 10D12) or purified Fab fragments (for 7B8 and 9G11) were incubated with the large trimeric SARS-CoV-2 S protein and the complexes were successfully resolved by Cryo-EM (Table 1). All three antibodies can bind to all three RBDs in the complex. Both 10D12 and 9G11 bind to RBD at up conformation, but the bindings have different angles. 10D12 was close to the pivotal axis, whereas 9G11 was close to the horizontal plane. Interestingly, we found that 7B8 bound not only with RBDs in all-up conformation (State 1) but also with RBD in down conformation (State 3, 1 RBD down, and 2 RBDs up), which was not seen for the other two antibodies, suggesting that 7B8 epitope remains accessible in down conformation (Fig. 2a; Supplementary Fig. S4). The folded RBD domain adopts a compact main body with a loop extending out from the top, resembling a clenched left hand with thumb up. Seventeen key residues at the RBD–hACE2 binding interface have been previously elaborated, encompassing two patches lining the upper palm-back ridge and a third patch at the thumb of the RBD domain^15^ (Supplementary Fig. S5). When we zoomed in on the single RBD-Fab region, the variable heavy–light chain (VH-VL) domains of 10D12 sit on the upper region of the palm side, and those of 9G11 sit on the upper region of the opposite side. 7B8 snaps at the thumb region of RBD, and all the contacting residues are within amino acid (AA)476–489 except P456 (Fig. 2b, c). We compared the structure-defined antibody epitopes with the structure of the RBD–hACE2 complex to determine how many residues in the epitopes lie at the hACE2 interface. For 10D12, 13 out of 24 residues were found to overlap with the hACE2 interface, which could explain a strong blocking effect. For 7B8, 4 out of 13 residues are the same as that of hACE2. For the 9G11 epitope, only Tyr449 is in the hACE2 interface, implying that significant hACE2 blocking was caused by minimal structural hindrance (Fig. 2d, e). The structure data validate our attempt to place three antibodies in the epitope binning. It provides direct evidence that antibody-mediated hACE2 blockade can be achieved by shielding different residues.

**Table 1:** Cryo-EM data collection, refinement, and validation statistics.

	S-10D12 (Ab1) consensus map (EMD-30981)	S-9G11 (Ab5) consensus map (EMD-30980)	RBD-10D12 (Ab1) focused refine (EMD-30979) (PDB 7E3C)	RBD-9G11 (Ab5) focused refine (EMD-30978) (PDB 7E3B)	S-7B8 (Ab4) consensus map (State 3) (EMD-30977) (PDB 7E39)	S-7B8 (Ab4) consensus map (State 1) (EMD-30976)
Data collection and processing						
Magnification	64k	64k	64k	64k	64k	64k
Voltage (kV)	300	300	300	300	300	300
Electron exposure (e^–^/Å^2^)	50	50	50	50	50	50
Defocus range (μm)	−1.4~−2.4	−1.4~−2.4	−1.4~−2.4	−1.4~−2.4	−1.4~−2.4	−1.4~−2.4
Pixel size (Å)	1.087	1.087	1.087	1.087	1.087	1.087
Symmetry imposed	C3	C3	C1	C1	C1	C1
Initial particle images (no.)	1,081,603	926,053	1,081,603	926,053	1,584,839	1,584,839
Final particle images (no.)	358,393	253,522	136,287	353,268	108,702	262,118
Map resolution (Å)	3.3	3.5	4.2	4.2	3.7	3.4
FSC threshold	0.143	0.143	0.143	0.143	0.143	0.143
Map resolution range (Å)	3.0-10.0	3.1-10.0	3.6-5.0	3.6-5.0	3.3-10	3.0-10
Refinement						
Initial model used (PDB code)			6ZER	6ZER	6ZER	
Map sharpening *B* factor (Å^2^)	−80.0	−129.2	−201.6	−258.3	−104.0	−90.0
Model composition						
Nonhydrogen atoms			2937	3299	3074	
Protein residues			405	421	414	
Ligands			1	1	1	
*B* factors (Å^2^)						
Protein						
Ligand						
R.m.s. deviations						
Bond lengths (Å)			0.006	0.005	0.004	
Bond angles (°)			0.803	0.703	0.720	
Validation						
MolProbity score			2.52	2.21	2.25	
Clashscore			20.86	15.93	15.80	
Poor rotamers (%)			0.00	0.00	0.00	
Ramachandran plot						
Favored (%)			82.12	92.37	90.15	
Allowed (%)			17.88	7.63	9.85	
Disallowed (%)			0.00	0.00	0.00

### Combination effect of neutralizing antibodies for viral mutants

**Fig. 3:**
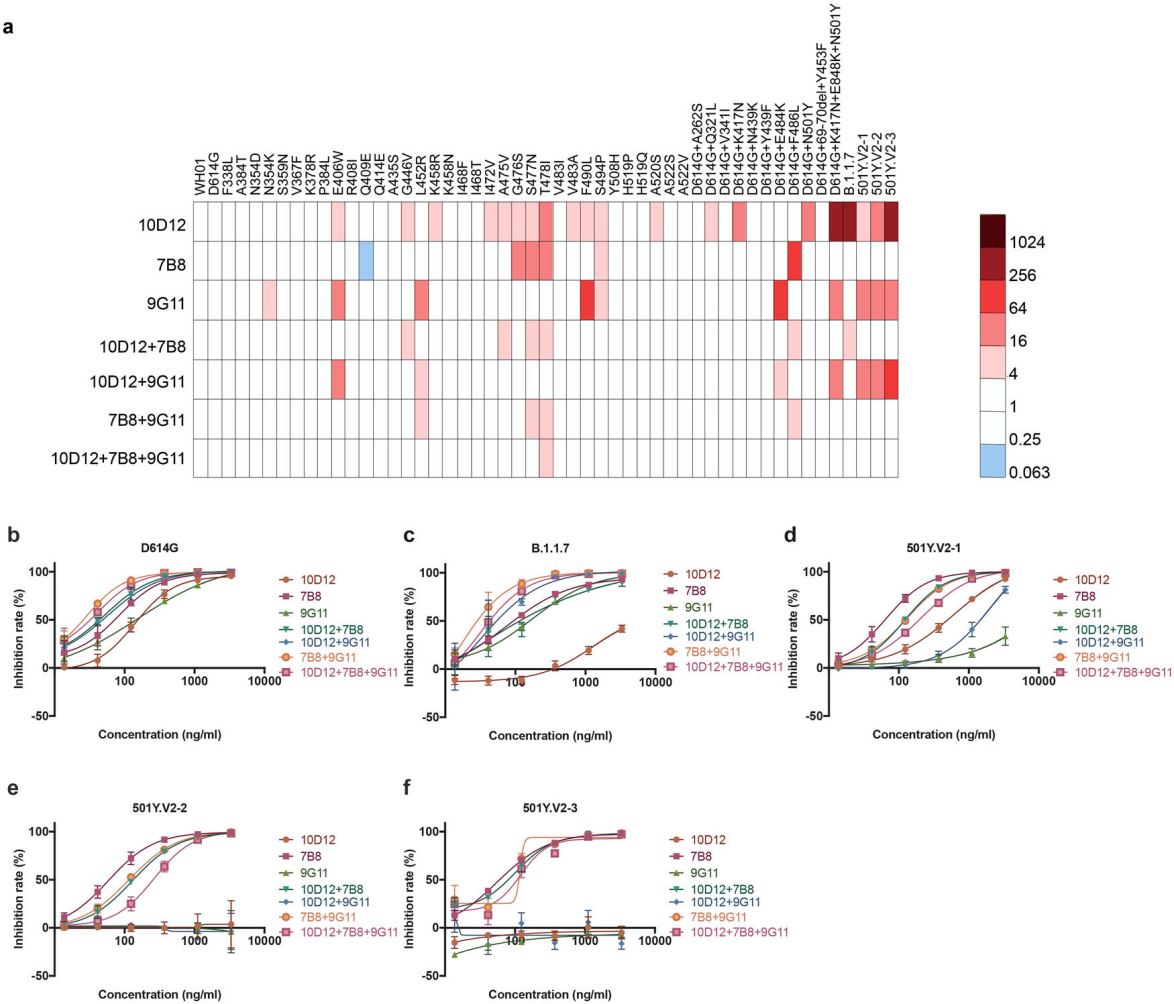
Analysis of the neutralizing activity of mAbs and their combinations on SARS-CoV-2 pseudotyped viruses with mutations in RBD. **a** Heatmap representation of neutralization reactions using three neutralizing mAbs and four cocktails against 51 pseudotyped viruses; the ratio of EC_50_ value (for each of the tested antibodies) detected for each of RBD-related mutant to the EC_50_ value for the reference Wuhan-1 (WH01) variant pseudotyped viruses. Light and dark represent decreased and increased viral resistance to mAb neutralization, respectively. The mixing ratio of different antibodies was 1:1 and 1:1:1, respectively. The antibody concentration was calculated according to the total antibody concentration after mixing. **b–f** Resistance of D614G mutant, B.1.1.7 mutant, and three 501Y.V2 mutant pseudotyped viruses to single or mixed antibodies.

**Fig. 4:**
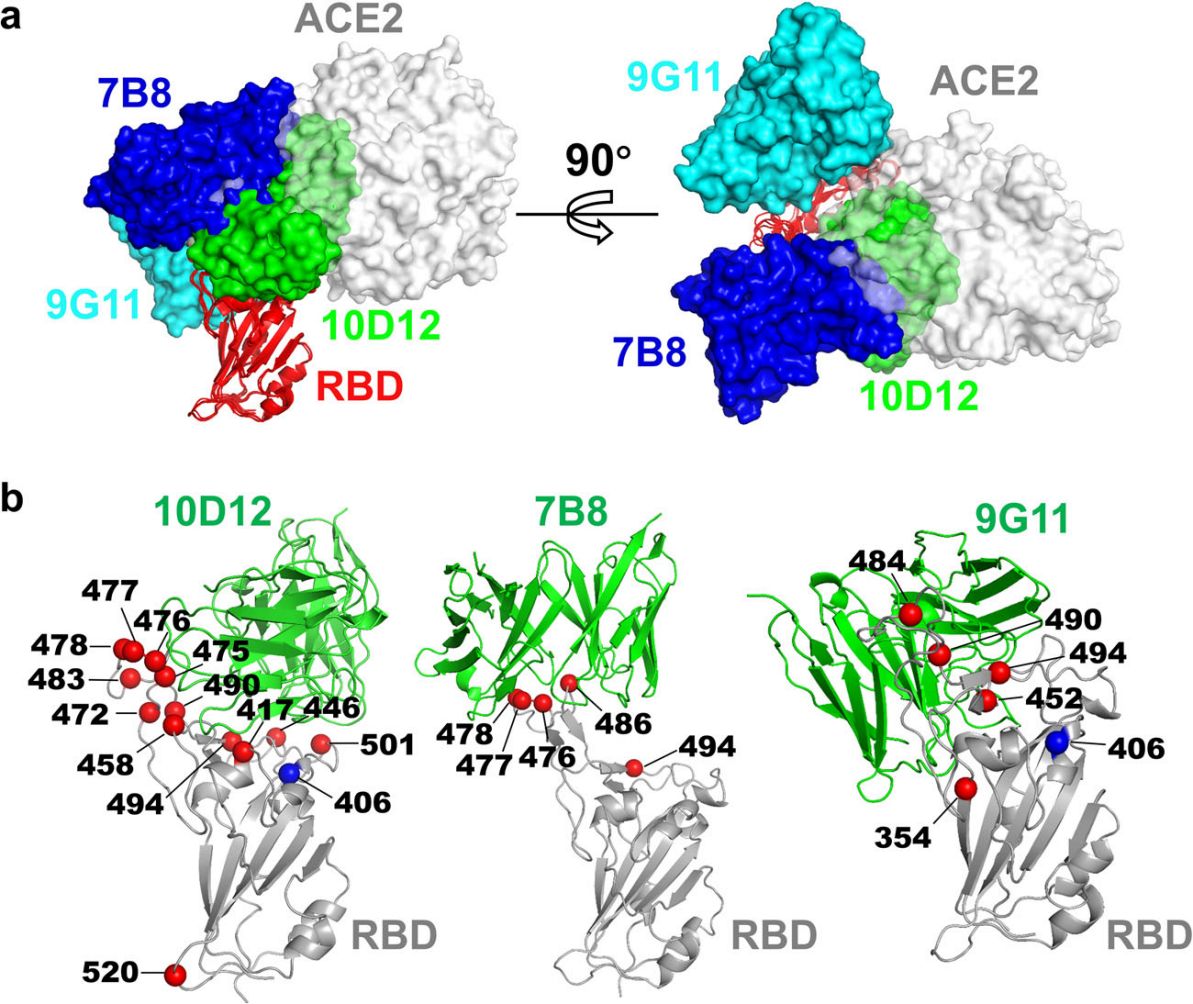
The structural basis for antibody cocktails to reduce the escape of viral mutants. **a** Variable regions of three selected neutralizing antibodies and hACE2 (PDB:6M0J) are shown as surfaces and superimposed based on RBD to demonstrate their relative positions and orientations. **b** The landscape of mutations in SARS-CoV2 RBD that affect the neutralizing activity of individual antibody clones. The residues that affected the neutralizing activity in Fig. 3 are shown as red spheres, except residue 406, which is highlighted as a blue sphere.

To investigate the neutralizing activity of three selected antibodies against SARS-CoV-2 mutants, we constructed 35 pseudotyped viruses with a single amino acid mutation in the RBD. Additionally, 15 pseudotyped viruses with combined mutations were generated, which carried similar mutations as circulating variants. The neutralizing activity of the mAb against the mutant pseudotyped virus was defined as an immune escape when its EC_50_ values increased over fourfold compared with that of WT SARS-CoV-2 (WH01). When we tested the three antibodies against the 50 mutant SARS-CoV-2 pseudotyped viruses, we found that the neutralizing activity of these antibodies decreased in varying degrees against the mutant variants. D614G variant was reported to have increased infectivity, but EC_50_ of all three antibodies remained unchanged. However, compared with the WT virus, the neutralizing activity of 10D12, 7B8, and 9G11 decreased significantly against 20, 5, and 10 of 50 variants, respectively (Fig. 3a–f). Most neutralization-resistant residues are at or very close to the Cyro-EM-defined structural epitope (Fig. 4b; Supplementary Fig. S6). Interestingly, some unexpected evasions were observed for a few variants. First, S494P is the only mutation found to have over fourfold resistance to all three mAbs. Serine 494 is near 10D12 and 9G11’s epitopes, but it is distant from the 7B8 epitope. Second, 10D12 showed reduced neutralizing against Q321L and A520S, both of which located at the base of RBD. Third, E406W drew intense attention recently, because it can escape both antibodies in the REGN-COV2 cocktail despite that it is not in the structural footprint of either antibody^11^. We also found that E406W mutation had increased resistance to both 10D12 and 9G11, but not to 7B8.

To enhance the neutralization potency of the antibodies, we mixed double or triple antibodies to make cocktails in the proportion of 1:1 or 1:1:1. Then 50 mutants were used to test the neutralizing activity of mAbs with different cocktail combinations. It was found that the cocktail strategy could expand the neutralization spectrum of the mAbs. The number of escaping variants decreased to 6, 7, 4, and 1 for 10D12 + 7B8, 10D12 + 9G11, 7B8 + 9G11, and 10D12 + 7B8 + 9G11, respectively. For the aforementioned S494P variant, the combination of any two antibodies prevented the escape. For the circulating B1.1.7 and three 501Y.V2 variants^16^, the neutralizing activity of 7B8 + 9G11 or 10D12 + 7B8 + 9G11 remained potent (Fig. 3a, b). Among the mAb combinations selected in this study, the broad-spectrum neutralization activity of cocktail antibodies is the Tri-combination, for which the only escape mutant was T478I. The T478I mutation accounted for 0.046% of circulating mutants, mainly found in Japan and England. The activity of 10D12 and 7B8 alone decreased by 35 times and 47 times against T478I mutant compared to WT. When the three antibodies were combined, the neutralizing activity decreased by 4.8-fold, just over the preset threshold of 4. Although the activity decreased, the incidence of EC_50_ still reached 0.195 μg/mL. Thus, in the 10D12 + 7B8 + 9G11 cocktail, antibodies can complement each other, resulting in the best broad neutralizing activity (Fig. 4a). For some variants, such as 501Y.V2, single antibody (7B8) may show higher potency than double- or triple-antibody combinations. It is due to the different starting concentrations: 10 μg/mL for the single 7B8 and with others; either 5 or 3.3 μg/mL in combination with one or two antibodies. No immune escape was observed for 501Y.V2 when 7B8 was employed, which showed the highest potency against 501Y.V2.

## Discussion

Since the outbreak of the pandemic COVID-19, intense attentions have been cast on neutralizing antibody or cocktails targeting the viral S protein. Most groups relied on PBMC from recovered COVID-19 individuals or related SARS patients as sources of antigen-specific B cells. Alternatively, a few groups showed that fully human neutralizing antibodies could be generated in humanized mouse models^6,9^. Using antibody repertoire-humanized mice instead of human subjects has two advantages. First, repetitive immunization with the defined viral immunogen, for example, RBD domain, in the presence of adjuvant is well accepted. Second, this procedure can be carried out with no need to access virus-infected individuals, enabling the scientific community to be prepared in advance against future threats. Given that spontaneous virus mutations arise frequently, it seems that any single neutralizing antibody clone is not enough to prevent all mutants. To increase the potency and to minimize mutant escape, a cocktail design combining two or more antibodies has been developed for the Ebola virus and SARS-CoV-2^17,18^. Biocytogen has recently developed a fully human antibody mouse termed RenMab, which to our knowledge is the only genome-edited model carrying the entire human variable region segments of heavy chain and kappa chain. We were interested in how divergent RBD-blocking epitopes can be and whether RenMab mice can generate high-quality fully human antibody cocktails. Several neutralizing antibody discovery campaigns were launched to increase the diversity by varying the immunogen formats and administration routes. In one of the campaigns, ten high-affinity blocking antibodies were discovered from SARS-CoV-2 RBD-immunized RenMab mice. Interesting patterns of V gene bias were observed. For example, IGHV3-66 is a dominant VH gene found at a frequency much higher than that in the normal VH composition, implying that it is positively selected. Unique repertoire pattern in human-derived neutralizing antibodies has been reported by many groups. Interestingly, these V genes (IGHV3-66 and IGHV3-53) contain NY and SGGS motifs in the germline sequence, which are important for antibody binding. The elegant study reported by the Regeneron group showed that many human antibody features, including variable‐diversity‐joining (VDJ) usage, CDR3 length, and heavy-light chain pairing, are well recapitulated in the repertoire of Velocimmune mouse^6^. Together, these studies support the values of humanized antibody mouse platforms in high-quality therapeutics discovery.

The extent of epitope overlapping for ten selected RBD-blocking antibodies was further studied by epitope binning. It turned out that there exist four distinct groups. Bin 1 encompasses most clones. The other three bins are nonoverlapping with each other. We were particularly interested in Bin 1, 3, and 4 since clones represented in these clusters showed more potent neutralizing activity. The antibodies in Bin 2, although binding to RBD at high affinity, had weaker activity. Thus, the representative clones from Bin 1/2/3 were studied by Cyro-EM in the antibody-spike complex and the data showed that they all contacted with the residues that mediate RBD-hACE2 binding (Fig. 2d).

Due to the strong specificity of mAb recognition, studies have shown that the neutralization activity of mAbs against SARS-CoV-2 mutants could be dramatically compromised, which may even result in their complete loss of neutralization activity^19–21^. Therefore, mutations of SARS-CoV-2 will be the biggest challenge of mAb therapy. In this study, the neutralization-resistant mutations in the RBD region are all related to the structure of 10D12, 7B8, 9G11, or the current popular RBD mutations. Among them, N501Y has the highest prevalence, exceeding 10.9% (N439K over 2.3%, L452R 0.49%, E484K 0.35%, Y453F 0.30%, K417N 0.19%, A520S 0.17%). D614G + K417N + E484K + N501Y is the representative mutation site of 501Y.V2 mutant, and its prevalence is more than 0.18%. D614G + 69-70Del + Y453F is a mink-related mutant with a prevalence of more than 0.30%, mainly in Denmark. The selected variants in this study are representative and can be used to evaluate the potency and spectrum of candidate therapeutic neutralizing antibodies in the current pandemic.

Through the combination of different cocktail strategies, we found that the cocktail of mAbs with distinct epitopes could significantly improve the broad-spectrum neutralization activity against different mutants. In the face of the present situation of SARS-CoV-2 mutation, cocktail mAb therapy will be the best choice. The 10D12 + 7B8 + 9G11 combination showed potent and broad-spectrum neutralization against circulating mutants, which is worthy of further clinical trials to verify the therapeutic efficacy. Some mAbs have unique neutralization mechanisms that can effectively improve their neutralization potency and spectrum, such as HB27 with double locking mechanism^22^, and P2C-1F11^23^ and H014^8^ involving much more amino acids in the binding interface of the RBD^24^. Noteworthily, any kind of mAbs, or even a combination of mAbs, may not necessarily satisfy neutralizing activity against all the mutants. Thus, naturally occurring mutations of SARS-CoV-2 should be closely monitored and investigated for the effect on the potency of therapeutics and vaccines. The design or combination of vaccines or mAbs should be adjusted according to the variation.

## Materials and methods

### Immunization

Fully human antibody mice recently developed by Biocytogen (RenMab) were used for protein immunization. In brief, 20 μg recombinant SARS-Cov-2 RBD (40592-V05H; Sino Biological) was emulsified in complete Freund’s adjuvant (CFA) and injected subcutaneously for the first immunization. Then mice were boosted with four additional immunizations in incomplete Freund’s adjuvant (IFA) every 14 days. Seven days after the fifth immunization, sera were collected and the antibody titer was determined by flow cytometry using CHO cells transfected with a plasmid encoding full-length SARS-CoV-2 S protein. Five mice with high levels of specific antibody titer were selected for hybridoma fusion.

### Hybridoma production and screening

Spleens were dissected and prepared for single-cell suspension. Antibody-secreting cells were enriched by negative selection of CD3^+^ T cells and IgM^+^ B cells on Miltenyi’s magnetic column. The flowthrough fraction was washed, counted, and fused with SP2/0 myeloma cells in the presence of 8 μg/mL polyethylene glycol derivatives (PEG, 03806; STEMCELL Technologies). After fusion, cells were seeded in 96-well plates and cultured at 37 °C. On day 10, the culture supernatant was collected and screened with a cell-based blocking assay. In brief, SARS-CoV-2 S-overexpressing CHO cells were first incubated with hybridoma supernatants for 30 min at 4 °C. Recombinant hACE2-human Fc fusion protein (10108-H05H; Sino Biological) was added and incubated for another 30 min. Fluorophore-conjugated secondary antibodies against antimouse IgG (115-606-071; Jackson ImmunoResearch) and antihuman IgG (109-116-098; Jackson ImmunoResearch) were stained to visualize the binding of hybridoma antibody and hACE2. Wells with apparent hACE blocking activity were chosen for subcloning on semisolid agar plates. Single clones were automatically picked using the Clonpix Image system (Molecular Devices).

### Preparation of fully human mAbs

To extract antibody variable region-coding sequences, hybridoma cells were lysed. mRNA was reverse transcribed to cDNA with SMARTer RACE Kit (Takara), followed by PCR with a common forward primer UPM-45 (CTAATACGACTCACTATAGGGCAAGCAGTGGTATCAACGCAGAGT) and heavy-chain reverse primer IgH-23-R(CTGGACAGGGATCCAGAGTTCCA) or kappa-chain reverse primer IgKC-tag (CTAACACTCATTCCTGTTGAAGCTCTTGAC). PCR products of the heavy chain and light chain were ligated into pEE12.4 and pEE6.4 expression vectors (Lonza Biologicals), respectively. Recombinant fully human IgG1 antibodies were produced using the transient expression system of ExpiCHO-S cells (Thermo Fisher) and antibodies were purified on AKTA Avant (GE healthcare) by protein A affinity chromatography.

### Surface plasmon resonance (SPR) analysis

To determine the binding affinity, purified antibodies were immobilized on a Protein A sensor chip (GE healthcare) to ~50 response units (RUs) using a Biacore 8 K (GE Healthcare) and a running buffer composed of HBS-EP+, pH7.4. Serial dilutions of WT SARS-CoV-2 RBD were flowed through the sensorchip system. The data were fitted into a 1:1 binding model using Biacore Evaluation Software (GE Healthcare). For epitope binning analysis, SARS-CoV-2 RBD was captured to ~50 RUs onto Biacore amine-coupled anti-His CM5 sensor surfaces. The first antibody was flowed onto the chip surface to reach a saturation level, followed by the flow of a second antibody into the system.

### S Protein expression and purification

S protein used in this study was prepared as previously described^25^. Briefly, the extracellular domain (ECD) (1–1208 aa) of S protein was cloned into the pCAG vector (Invitrogen) with two proline substitutions at residues 986 and 987, a “GSAS” substitution at residues 682–685, and a C-terminal T4 fibritin trimerization motif followed by one Flag tag, and the construct was overexpressed in HEK 293 F cells. S-ECD secreted into the medium was purified by one step of anti-Flag affinity purification and size-exclusion chromatography.

### Cryo-EM sample preparation

Six microliters purified S-ECD at the concentration of 2 mg/mL was incubated with 3 μL full-length IgG 10D12 at the concentration of 2.3 mg/mL on ice for 1 min and then the mixture was applied to prepare cryo-EM grids. For 9G11 and 7B8, 6 μL S-ECD was incubated with a 5 μL Fab fragment of the antibodies at the concentration of 1.2 mg/mL on ice for 1 min. For cryo-EM, frozen-hydrated specimens were prepared with a Thermo Fisher Vitrobot Mark IV plunger. S-antibody complex of volume 3.5 μL was placed on a glow discharged holey carbon grid (Quantifoil Au R1.2/1.3). The excess solution from the grid was blotted for 2.5–3.0 s at 100% humidity at 8 °C before the grid was flash frozen in liquid ethane cooled at liquid-nitrogen temperature.

### Cryo-EM data collection

Cryo-EM data were collected on a Thermo Fisher Titan Krios G3i electron microscope equipped with a Gatan K3 direct electron counting camera (Supplementary Fig. S7). The microscope was operated at 300 kV, and images of the specimen were recorded with a defocus range from –1.4 to –2.4 µm at a calibrated magnification of 64k× in super-resolution mode with a pixel size of 0.54 Å. The movie stacks, each containing 32 subframes, were recorded with the semiautomated low-dose acquisition program EPU, with a total accumulated dose of approximately 50 electrons/Å^2^.

### Cryo-EM data processing

The raw super-resolution dose-fractionated image stacks were 2× Fourier binned, aligned, dose-weighted, and summed using MotionCor2^26^, resulting in summed micrographs in a pixel size of 1.087 Å. Contrast transfer function (CTF) parameters were estimated using CTFFIND4.1^27^. All the following processing steps were performed in RELION 3.1^28^. First Laplacian-of-Gaussian method was used to pick particles automatically. Then all these particles were subjected to several rounds of reference-free 2D classification to remove contaminants and bad particles. After that 3D classification was performed using a map derived from the PDB model as the initial reference model. The most homogeneous particles were selected for the final 3D auto-refinement with C3 symmetry (10D12 and 9G11) or without symmetry (7B8). For the 7B8–S complex, 3D classification yielded two good classes (State 1 and State 3) and we reconstructed both structures. To improve the map quality of the interface between RBD and antibody 10D12 or 9G11, the dataset was expanded with C3 symmetry and then subjected to focused 3D classification and then a final round of focused 3D auto-refinement (Supplementary Fig. S8). Reconstruction resolutions were determined based on the gold-standard Fourier shell correlation (FSC = 0.143) criterion^29^.

Cryo-EM data collection, refinement, and validation statistics could be found in Table 1.

### Site-directed mutagenesis

Based on pcDNA3.1.S2^30^, 50 mutant plasmids were generated^10,24^. Briefly, the codon-optimized S gene of SARS-CoV-2 (GenBank: MN_908947) was inserted into pcDNA3.1 recombinant plasmid named pcDNA3.1.S2^30,31^. The pcDNA3.1.S2 recombinant plasmid was used as the template to generate the plasmid with mutagenesis in the S gene. Following the procedure of circular PCR, 15–20 nucleotides before and after the target mutation site were selected as forward primers, while the reverse complementary sequences were selected as reverse primers. Following site-directed mutagenesis PCR, the template chain was digested using DpnI restriction endonuclease (NEB, USA). Afterward, the PCR product was directly used to transform *E. coli* DH5a competent cells; single clones were selected and then sequenced. The primers designed for the specific mutation sites are listed in Supplementary Table S2.

### Preparation of pseudotyped viruses

Pseudotyped viruses were constructed according to our previous study^30,31^. The Huh-7 cells were digested and adjusted to a concentration of 5–7 × 10^5^ cells/mL the day before transfection. Then, cells in 15 mL of medium were transferred to a T75 cell culture flask and incubated overnight at 37 °C in an incubator with 5% CO_2_. When the cells reached 70%–90% of confluence, the medium was discarded and 15 mL G*ΔG-VSV virus (vesicular stomatitis virus G pseudotyped virus, Kerafast) at a concentration of 7.0 × 10^4^ median tissue culture infective dose per milliliter (TCID_50_/mL) was used for infection. At the same time, the cells were transfected with 30 μg S protein expression plasmid according to the user manual and then cultured in an incubator with 5% CO_2_ at 37 °C. The cell supernatant was discarded after 6–8 h and the cells were gently rinsed twice with PBS + 1% FBS. Next, 15 mL of fresh complete DMEM was added to the T75 cell culture flask, and after 24 h of culture in an incubator at 37 °C and 5% CO_2_, the supernatant containing pseudotyped virus was harvested, filtered, aliquoted, and frozen at –70 °C for further use.

### Neutralization assay

The virus neutralization assay was conducted as described previously^30,31^. Briefly, 100 μL serial dilutions of mAb preparations were added into 96-well plates. After that, 50 μL pseudoviruses with a concentration of 1200 TCID_50_/mL were added to the plates, followed by incubation at 37 °C for 1 h. Afterward, Huh-7 cells were added to the plates (2–3 × 10^4^ cells/100 μL cells per well), followed by incubation at 37 °C in a humidified atmosphere with 5% CO_2_. Chemiluminescence detection was performed after 24 h of incubation using a luminometer (PerkinElmer, Ensight). Positive was determined to be 500-fold higher than the negative (cells only) in terms of relative luminescence unit (RLU) values. The Reed–Muench method was used to calculate the virus neutralization titer (EC_50_)^31^. The results are based on three replicates.

## Supplementary information

Supplementary material

## Data Availability

Six cryo-EM reconstructions and three corresponding atomic models for antibody-S complexes have been deposited in EMDB and PDB, respectively, under accession numbers listed in Table 1.

## Materials availability

Correspondence and request for materials should be addressed to Y.W. or W.H.
