# Supplementary material for: Three epitope-distinct human antibodies from RenMab mice neutralize SARS-CoV-2 and cooperatively minimize the escape of mutants
Source: Cell Discov. 2021 Jul 20;7:53. doi: 10.1038/s41421-021-00292-z (PMC8290868; doi:10.1038/s41421-021-00292-z)
Supplement: Supplementary file 1 — Supplementary material [file 41421_2021_292_MOESM1_ESM.pdf]

Supplementary materials

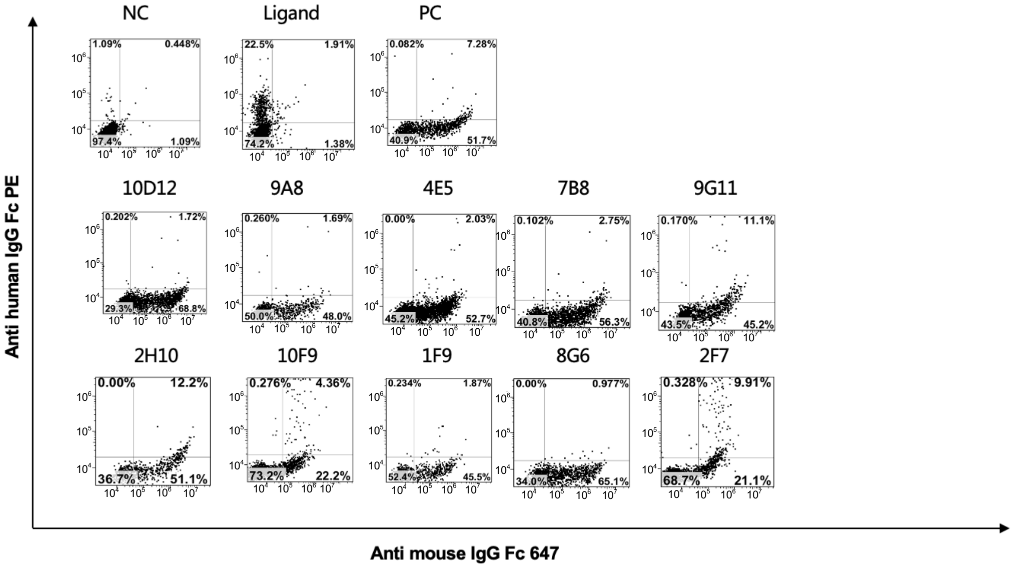

Supplementary Fig. S1 Screening of hybridoma clones from SARS-CoV-2 RBD immunized RenMab mice. SARS-CoV-2 spike-expressing CHO cells were incubated with culture supernatant of hybridoma clones and recombinant hACE2-Fc. Cells were washed and stained with fluorophore-conjugated secondary antibodies to show the binding of mouse hybridoma antibody and hACE2. PC is the post-immune sera collected from RBD-immunized animals.

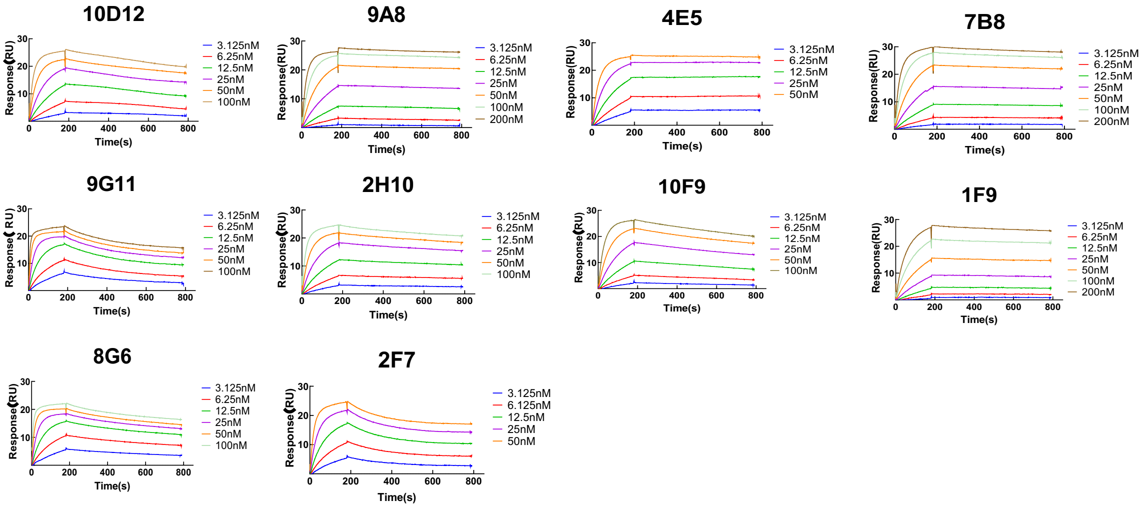

Supplementary Fig. S2 SPR analysis of SARS-CoV-2 RBD binding affinity. Recombinant RBD-specific hIgG1 antibodies were immobilized on protein A biosensor. Serial dilution of SARS-CoV-2 RBD flew over the biosensor.

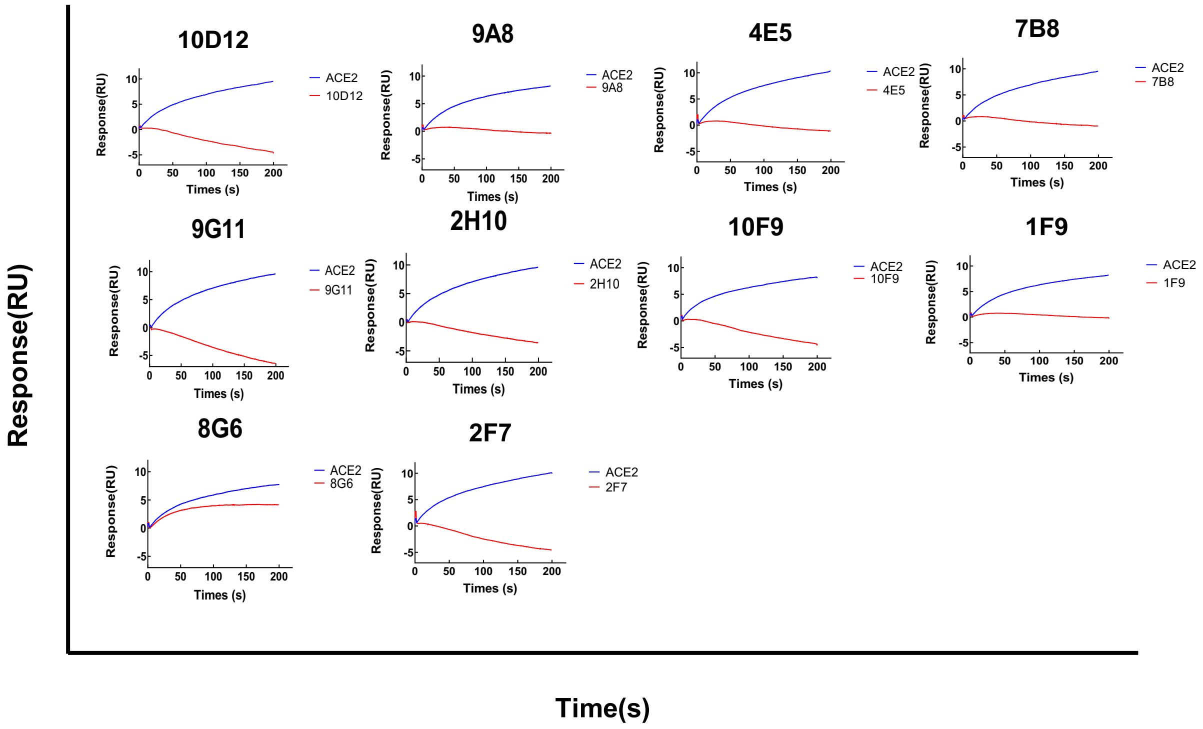

Supplementary Fig. S3 SPR analysis of hACE2 blocking activity. SARS-CoV-2 RBD was first immobilized on CM5 chip. Then hACE2 flowed over the biosensor surface in the presence (red) or absence (blue) of the indicated blocking antibody.

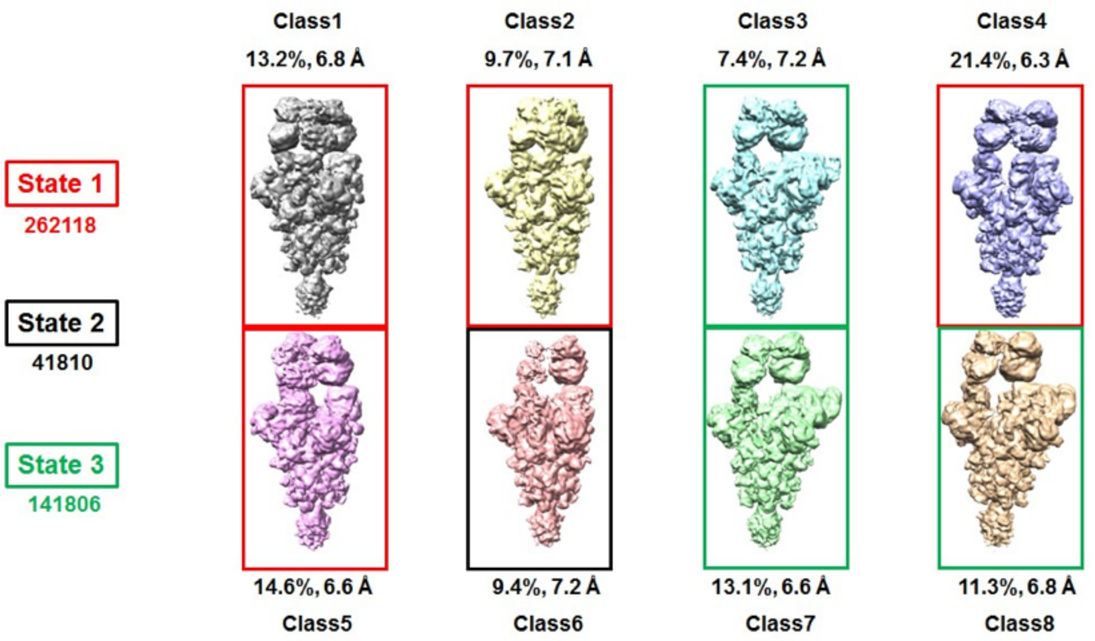

Supplementary Fig. S4 3D classification of 7B8-S complex revealed three different states. State 1 (3 RBDs up and bound with 7B8), State 2 (2 RBDs up and bound with 7B8, 1 RBD down without 7B8 binding) and State 3 (2 RBDs up and 1 RBD down, and all bound with 7B8). Particles belonging to State 2 were not good enough for further 3D refinement, so only high-resolution reconstructions of State 1 and State 3 were described in this study.

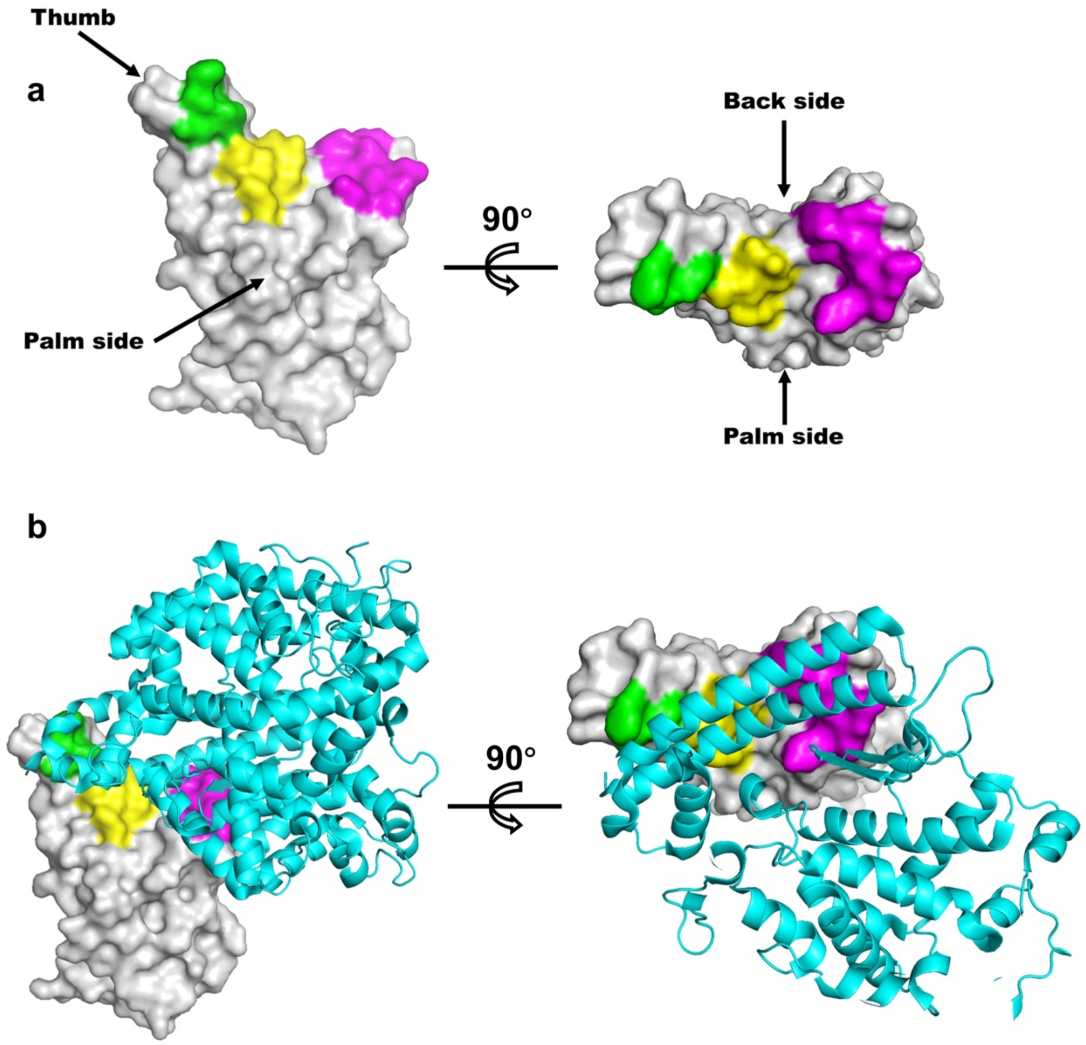

Supplementary Fig. S5 ACE2 interaction is mediated by three patches on SARS-CoV-2 RBD. a The overall structure of RBD domain resembles a clenched left hand with thumb up. The seventeen key residues at the ACE interface can be grouped to three patches. The green patch includes residues A475, F486, N487, C488 and Y489. The yellow patch includes residues K417, Y453, L455, F456 and Q493. The magenta patch includes residues G446, Y449, G496, Q498, T500, N501, G502 and Y505. **b** ACE2 (cyan) binds with RBD via these three patches.

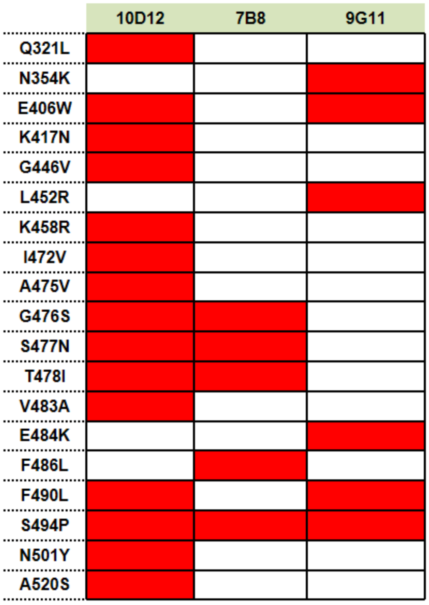

Supplementary Fig. S6 The list of identified amino acid mutations that affected three neutralizing monoclonal antibodies. Red boxes indicate that the mutations were found to reduce neutralizing activity over 4-fold.

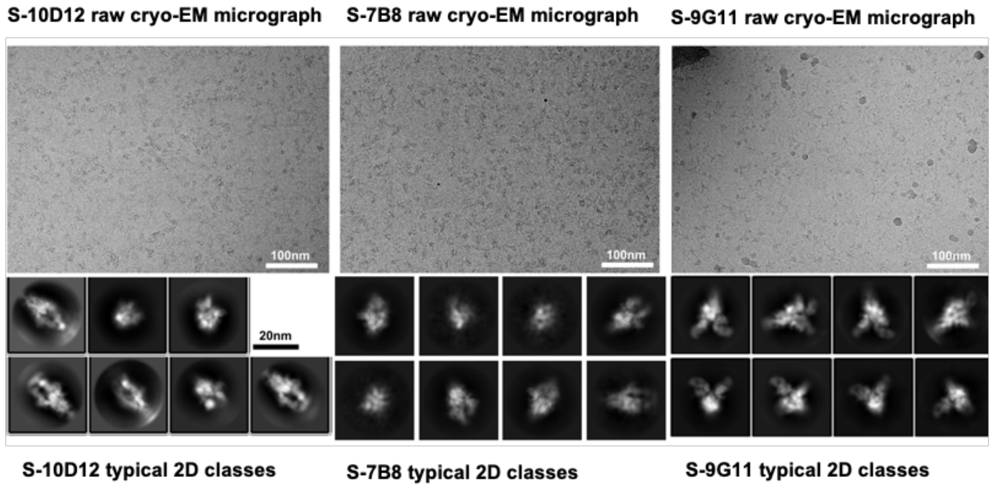

Supplementary Fig. S7 The raw cryo-EM micrographs (upper panel) and typical 2D class averages (lower panel) of S-10D12/7B8/9G11 complexes.

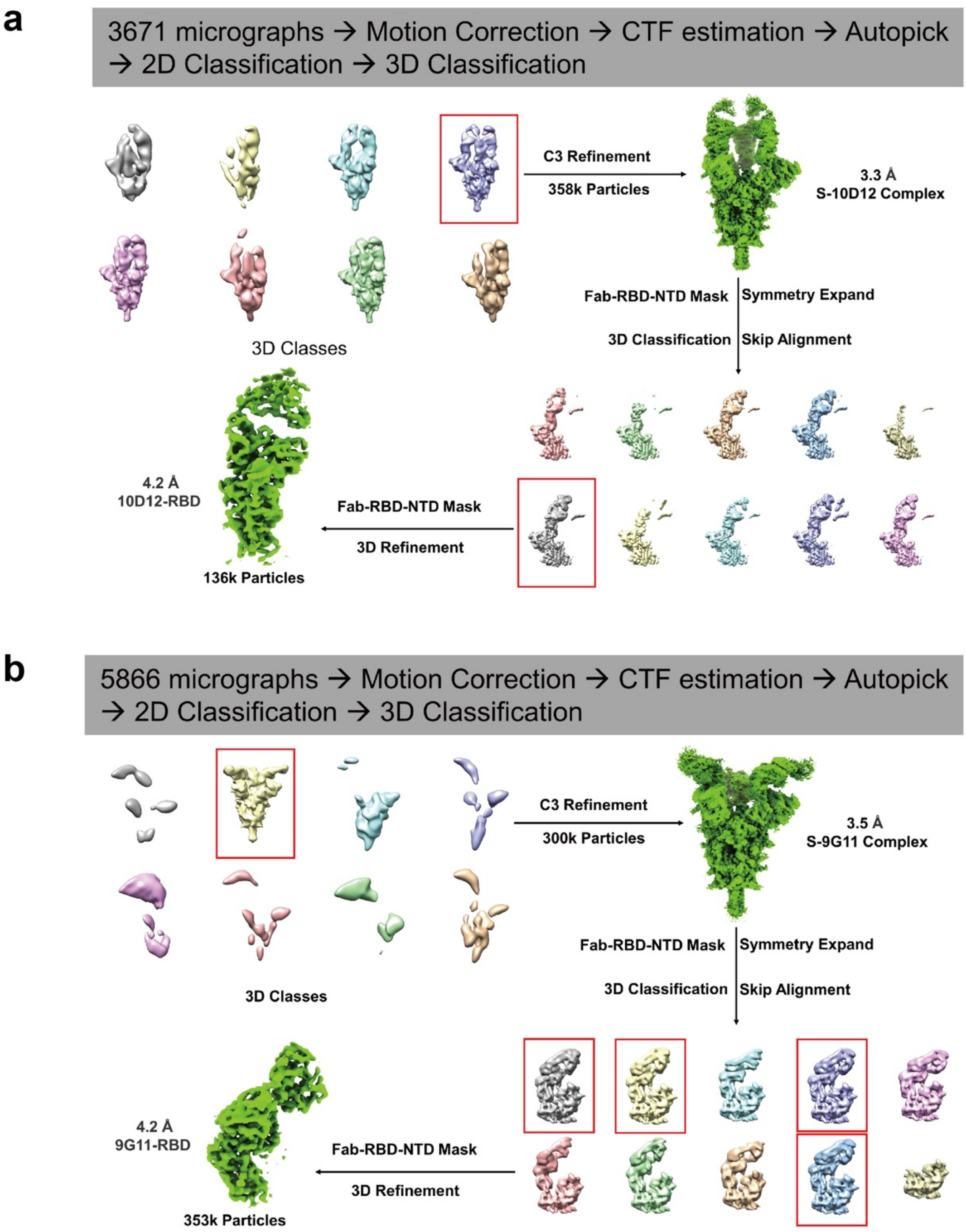

Supplementary Fig. S8 Cryo-EM data processing of the S-10D12 (a) and S-9G11 (b) complexes. The processing of S-7B8 is similar so the workflow for S-7B8 is not shown.

Supplementary Table S1. Summary of Top 10 clones

Clone ID	Epitope bin	Heavy chain V gene	H-CDR3	Kappa chain V gene	L-CDR3	ka(1/Ms)	kd(1/s)	kD(M)
10D12	B1	IGHV3-53*04	CARDLDYYGMDVW	IGKV1-9*01	CQQLNSYPPLTF	3.14E+05	4.80E-04	1.53E-09
2H10	B1	IGHV3-66*01	CARDLDYYGMDVW	IGKV1-9*01	CQHLNSYPPTF	3.14E+05	2.58E-04	8.21E-10
10F9	B1	IGHV3-66*01	CARDLLVYGMDVW	IGKV1-9*01	CQQLNSYPPTF	2.61E+05	4.98E-04	1.91E-09
9A8	B1	IGHV3-66*01	CAREVVGSNSNMDVW	IGKV1-33*01	CQQYDNLPRTF	3.56E+05	7.22E-05	2.03E-10
1F9	B1	IGHV3-23*01	CARDRKLLYFFDYW	IGKV1-33*01	CQQYDHLPLTF	8.99E+04	9.88E-05	1.10E-09
4E5	B2	IGHV4-31*02	CVSDRLDPYNFGMDVW	IGKV1-33*01	CQQYDNLPYTF	6.45E+05	2.46E-07	3.82E-13
2F7	B2	IGHV4-31*02	CARDRLDPYNFGMDVW	IGKV1-33*01	CQQYHNLPYTF	1.18E+06	7.90E-04	6.72E-10
7B8	B3	IGHV3-33*01	CARETVSYGMDVW	IGKV1D-17*01	CLQHNSYPYTF	1.82E+05	9.55E-05	5.23E-10
9G11	B4	IGHV4-59*01	CARDRGYSSGW	IGKV1-33*01	CQQYDNIPFTF	6.28E+05	8.74E-04	1.39E-09
8G6	B4	IGHV4-39*01	CVRQANWGSFDYW	IGKV1-33*01	CQQYDNLLMWTF	6.84E+05	5.51E-04	8.05E-10

Supplementary Table S2. Primers used for site-directed mutagenesis.

Name		Sequence (5'-3')
Epitope related mutagenesis	Q321L-F	CTACCAGACCAGCAATTTCAGAGTGCTACCTACCGAGAGCATCGTGAG
	F338L-F	TATCACCAATCTGTGCCCTCTGGGCGAGGTGTTCAATGCCACC
	V341I-F	ACCAATCTGTGCCCTTTCGGCGAGATCTTCAATGCCACCAGATTCGCCAG
	A348T-F	AGGTGTTCAATGCCACCAGATTCACCAGCGTGTACGCATGGAACCGCAAG
	N354D-F	GATTCGCCAGCGTGTACGCATGGGACCGCAAGCGGATAAGCAATTGCG
	N354K-F	AGATTCGCCAGCGTGTACGCATGGAAGCGCAAGCGGATAAGCAAT
	S359N-F	CGCATGGAACCGCAAGCGGATAAATAATTGCGTGGCCGACTACAGCGTGC
	V367F-F	ATTGCGTGGCCGACTACAGCTTCCTGTACAATAGCGCCAGCTTC
	K378R-F	ATAGCGCCAGCTTCAGCACCTTCCGATGTTATGGTGTTTCGCCAACAAAG
	P384L-F	CCTTCAAATGTTATGGTGTTTCGCTGACAAAGCTGAATGACCTGTGCT
	R408I-F	ATCAGAGGCGACGAGGTGATACAGATCGCGCCAGGGCAG
	Q409E-F	AGAGGCGACGAGGTGAGAGAGATCGCGCCAGGGCAGACCGGCA
	Q414E-F	TGAGACAGATCGCGCCAGGGGAGACCGGCAAGATCGCCGACTACAAT
	A435S-F	CGACTTCACCGGCTGCGTGATCAGCTGGAACTCTAACAACCTGGACTC
	N439K-F	CTGCGTGATCGCGTGGAACTCTAAGAACCTGGACTCGAAAGTTGGAGGC
	G446V-F	ACCTGGACTCGAAAGTTGTGGGCAATTACAATTACCTGT
	L452R-F	GTTGGAGGCAATTACAATTACCGATACAGACTGTTCAGAAAGAGCAAT
	K458R-F	CAATTACCTGTACAGACTGTTCAGACGTAGCAATCTGAAGCCTTTCGAG
	K458N-F	CAATTACCTGTACAGACTGTTCAGAAATAGCAATCTGAAGCCTTTCGAG
	I468F-F	AATCTGAAGCCTTTCGAGAGAGACTTTAGCACCGAGATCTACCAGGCCGG
	I468T-F	CAATCTGAAGCCTTTCGAGAGAGACACCAGCACCGAGATCTACCAGGCCG
	I472V-F	TTCGAGAGAGACATCAGCACCGAGGTGTACCAGGCCGGCAGCACACCGTG
	A475V-F	GACATCAGCACCGAGATCTACCAGGTGGGCAGCACACCGTGTAATGGCGT
	G476S-F	TCAGCACCGAGATCTACCAGGCCAGCAGCACACCGTGTAATGGCGTGGA
	T478I-F	ATCTACCAGGCCGGCAGCATCCCGTGTAATGGCGTGGAGGGC
	V483A-F	AGTAGCAATTGAAGCCCTCTGCGCCATTACACGGTG
	V483I-F	CACCGTGTAATGGCATCGAGGGCTTCAATTGCTACTTCCC
	F490L-F	CGTGGAGGGCTTCAATTGCTACCTGCCTCTGCAGAGCTACGGCTTCCAG
	Y508H-F	ACCAATGGCGTGGGCTACCAGCCTCACAGAGTGGTGGTGCTGAGCTTCG
	H519P-F	GGTGGTGCTGAGCTTCGAGCTGCTGCCTGCTCCCGCTACCGTGTGCGGCC
	H519Q-F	GGTGGTGCTGAGCTTCGAGCTGCTGCAGGCTCCCGCTACCGTGTGCGGC
	A520S-F	TGCTGAGCTTCGAGCTGCTGCACAGCCCCGCTACCGTGTGCGGCCCTA
	A522S-F	GCTTCGAGCTGCTGCACGCTCCCAGCACCGTGTGCGGCCCTAAGAAGAG
	A522V-F	GCTTCGAGCTGCTGCAGAGCCCCGTGACCGTGTGCGGCCCTAGAAGA
	D614G-F	GTGGCCGTGCTGTACCAGGGCGTGAATTGCACCGAGGT
Mink related mutagenesis	69-70del-F	GTGACCTGGTTCCACGCCATCAGCGGCACCAATGGCACCAAGAG
	A262S-F	GGACCGCTGGAAGCGCGGCATATTACGTGGGCT
	Y453F-F	GTTGGAGGCAATTACAATTACCTGTTCAGACTGTTCAGAAAGAGCAATC

	F486L-F	GTAATGGCGTGGAGGGCCTGAATTGCTACTTCCCTCTGC
501Y.V2 related mutagenesis	L18F-F	TGGTGAGCAGCCAGTGCGTGAATTTCACCACCAGAACCCAGC
	D80A-F	CAATGGCACCAAGAGATTCGCCAATCCTGTGCTGCCTTTCAAT
	D215G-F	CACCCATTAATCTGGTGAGAGGCCTGCCTCAGGGCTTCAGC
	LAL242-244Del-F	TATCACCAGATTCCAGACCCTGCACAGATCATATCTTACACC
	K417N-F	CGCCAGGGCAGACCGGCAATATCGCCGACTACAATTAC
	E484K-F	CACCGTGTAATGGCGTGAAGGGCTTCAATTGCTACTTCC
	N501Y-F	AGAGCTACGGCTTCCAGCCTACCTACGGCGTGGGCTACCAGCCTTACAG
	A701V-F	CTACACCATGAGCCTGGGCGTGGAGAATAGCGTGGCCTAC
B.1.1.7 related mutagenesis	145del	GACCCTTTCCTGGGTGTTTATCATAAGAACAACAAGAGCTGGATGG
	A222V	ACCTGCCTCAGGGCTTCAGCGTGCTGGAGCCTCTGGTGGACCTG
	S477N	GCACCGAGATCTACCAGGCCGGCAACACACCGTGTAATGGCGTGGAGGGC
	A570D	CAACAATTCGGCAGAGACATCGACGACACCACAGATGCTGTAAGAGAC
	P681H	CTACCAGACCCAGACCAATAGCCACAGAAGAGCCAGAAGCGTGGCCAGCC
	T716I	CAATAATAGCATCGCCATCCCTATCAATTTCACCATCAGCGTGACCAC
	S982A	TACTCAACGACATCCTGGCGAGACTGGACAAGGTGGAGGCCGA
	D1118H	ACGAGCCTCAGATCATCACCACCCACAATACCTTCGTGAGCGGCAA
